# Synthetic osteobiologics in spine surgery: a review

**DOI:** 10.55730/1300-0144.5941

**Published:** 2024-10-25

**Authors:** Aydın Sinan APAYDIN, Eli JOHNSON, Prince ANTWI, Brett ROCOS, Michael M. HAGLUND, Christopher I. SHAFFREY, Peter PASSIAS, Muhammad M. ABD-EL-BARR, Khoi THAN

**Affiliations:** 1 Department of Neurosurgery, Faculty of Medicine, Karabük University, Karabük, Turkiye; 2 Department of Neurosurgery, Faculty of Medicine, Duke University, Durham, NC, USA

**Keywords:** Synthetic osteobiologics, spine fusion, osteobiologics

## Abstract

Osteobiologics are increasingly used in orthopedics and spine surgery to facilitate bone healing and prevent nonunion. Synthetic osteobiologics are artificial materials crafted in laboratories that aim to replicate the natural composition and functionality of bone. Notable materials such as calcium phosphate and calcium sulfate are engineered to mirror the mineral aspect of bone. They mimic human bone functionality, exhibiting osteoconductive, osteoinductive, and osteogenic properties. These characteristics promote cell attachment, migration, recruitment, and differentiation. Consequently, synthetic osteobiologics (osteoconductive grafts) have been introduced in bone fracture repair. The main strength of synthetic osteobiologics in spine surgery lies in improving fusion rates and clinical outcomes. The commercial biologics landscape boasts an excess of 350 bone substitute materials, a number that continues to grow exponentially with the development of subtypes. However, the proliferation of these products, primarily driven by the medical device industry and nonacademic entities, has been accompanied by a significant dearth of supporting empirical data. This deficiency underscores the imperative need for rigorous scrutiny and research to establish a solid foundation for their utilization. Healthcare professionals require high-quality research in large prospective studies with satisfactory follow-up periods to interpret and compare the performance of osteobiologics. It is particularly imperative to study the added cost of using these materials in spine surgery. In the current review, we provide an overview of the currently available synthetic osteobiologics used in spine surgery.

## 1. Introduction

Spine surgery has been continuously progressing, with advances over the last 10 years resulting in numerous surgical approaches to the spine, the development of minimally invasive techniques, the development of new spinal instrumentation, and the introduction of biologics to promote spinal fusion. Natural osteobiologics are obtained from a patient (autograft), donor (allograft), or animal sources (xenograft). Synthetic osteobiologics are man-made materials designed in laboratories. They are designed to mimic native bone structure and function. Examples include calcium phosphate and calcium sulfate, which are designed to resemble the mineral components of bone. Osteobiologics aid in averting local complications associated with harvesting autografts, such as pain, hematoma, and infection. Osteobiologics used in spine surgery include mineralized allografts, demineralized bone matrices, cellular-based matrices, growth factors, and synthetics. For example, bone morphogenetic proteins (BMP), platelet-rich plasma (PRP), calcium phosphate cements, bioactive glass, and beta-tricalcium phosphate (B-TCP) are all products used to stimulate bone formation. In this study, we review synthetic osteobiologics currently employed in spine surgery, discuss their mechanisms of action, and analyze the available data on the fusion rates and related complications.

## 2. Synthetic osteobiology

The study of synthetic materials for various applications has significantly advanced in recent years in efforts to reduce the reliance on autograft bone. Advances in materials science and biomedical engineering have laid the groundwork for a promising future where synthetic substitutes could surpass the performance and cost-effectiveness of allograft materials. This movement marks an evolution in medical material innovation, where synthetic substitutes are not merely matching but potentially exceeding the capabilities of conventional biological materials [1].

Currently, there is a diverse range of synthetic products, each with distinct biomechanical properties that dictate how well these materials can withstand physical stresses and strains. Additionally, their biodegradability varies, affecting how they interact with biological systems over time. Further variation lies in their microscale architecture, encompassing fine structural details that influence their performance and integration with biological tissues. Lastly, these synthetic products also differ in the properties that affect their handling during surgical procedures, including ease of use and adaptability to different surgical techniques. This variety offers a range of options for different medical applications, highlighting the advancements and customization possibilities in synthetic material technology [2].

To categorize the bone regenerative capabilities of various grafts, four key characteristics are typically considered: osteogenesis, osteoinductivity, osteoconductivity, and osteointegration [3]. Osteogenesis is the term used to denote the development of bones. This process involves osteoinductivity, an active mechanism where bone formation is governed and directed by osteogenic signaling pathways. In contrast, osteoconductivity refers to a passive process, where bone growth is facilitated along either the surface or within the internal structure of a material. Osteointegration occurs when bone growth effectively creates a stable linkage between a product’s surface and the bone tissue. Each of these characteristics must be considered when selecting materials for bone repair and regeneration.

Three primary classifications of synthetic osteobiologics are described: ceramics, bioactive glasses, and polymer-based materials. Ceramics are used for bone substitution due to their osteoconductive properties [4]. Bioactive glasses are used for their ability to bond with bone tissue, promoting healing [5]. Polymer-based materials are frequently employed in complex bone defects due to their versatility and moldability [6]. These scaffolds are structurally designed to mimic native bone tissue architecture, which is important for their effectiveness. These materials may be utilized independently, each offering distinct advantages for specific clinical scenarios. However, the recent trend towards developing composites as combinations of these substances has opened new avenues in biomaterials research [2]. Composites leverage the strengths of each constituent material, resulting in enhanced biomechanical properties, improved biodegradability, and tailored microscale architectures. This innovative approach allows for the creation of biomaterials with unique properties tailored for specific applications in bone repair and regeneration.

Following the use of osteobiologics in implant dentistry, these materials have become central to bone fusion, leading to an explosion in the variety of available materials. Despite this, there is limited evidence supporting their specific uses in spine surgery, and it is imperative to accurately and reliably understand the outcomes associated with their use [7]. Within the limits of currently available data, synthetic osteobiologics are associated with improved fusion rates and clinical outcomes in spine surgery [8].

There are several osteobiological compounds available on the market, each with distinct biochemical properties, osteogenic mechanisms, and patient-reported outcomes (Table). For this review, we conducted a systematic search of the literature using the PubMed, Web of Science, and Embase databases. Search queries using specific keywords including “osteobiologics,” “spine surgery,” “clinical outcomes,” and “biomechanical outcomes” were used. Inclusion criteria were as follows: studies in the English language involving human subjects who underwent spine surgery. However, studies reporting in vitro biomechanical outcomes were also included to broaden the scope while maintaining a focus on clinical patient-centered data and studies presenting robust clinical data from retrospective or prospective clinical research, ensuring that the included studies demonstrated clinical efficacy and applicability in contemporary spine surgery [8]. This review mainly concentrates on clinical outcomes following spine surgery; studies reporting applications of osteobiological compounds in procedures other than spine surgery have been excluded, as have case reports.

### 2.1. Vitoss synthetic cancellous bone

As an enhanced version of B-TCP, Vitoss (Stryker, Kalamazoo, MI, USA) is a low-density, porous construct made by fusing nanoparticles with diameters of approximately 100 nm. Utilizing particles at the nanoscale enhances the structure’s microporosity, resulting in a scaffold that is 90% linked with pore sizes ranging from 1 to 1000 μm. The rapid resorption of the scaffold, in conjunction with the rate of new tissue ingrowth, is due to its surface exposure to the biological milieu. Vitoss has a chemical composition similar to that of native bone, which contains approximately 35% calcium, 15% phosphorous, and 4.5% carbonate by weight in a Ca/P molar ratio of 1:71. Vitoss contains 39% calcium and 20% phosphorous, with a ratio of approximately 1:5 [9].

B-TCP has a distinctive basophilic reticular structure. Because the resorption of B-TCP by dissolution occurs concurrently with osteogenesis, it appears that bone replaces the B-TCP [9]. The dissolution rate of B-TCP in saline, simulating body fluids, exceeds that of hydroxyapatite (HA). Consequently, B-TCP constructs tend to disintegrate more readily, leading to the formation of small particles that are subsequently absorbed by phagocytes. Under acidic conditions, various cell types, such as monocytes, fibroblasts, osteoclasts, multinucleated cells, and macrophages, play a role in the biodegradation of the calcium phosphate implant without eliciting a significant inflammatory response. This limited inflammatory response offers a significant advantage and highlights the biocompatibility of B-TCP in biological environments [9]. As a synthetic bone graft substitute, Vitoss provides an osteoconductive scaffold for bone regeneration, with a composition and structure designed to promote rapid integration and resorption.

Vitoss is recommended for the treatment of osseous deformities, particularly those resulting from trauma. It is available in various forms, including blocks, morsels, moldable packs, malleable strips, and foam packs, which can be paired with autogenous blood or bone marrow. Designed for filling bone voids and gaps in the skeletal system, including the spine, pelvis, and extremities, Vitoss is intended for bony injuries that would not heal independently [9]. Vitoss offers several benefits, such as a longer shelf life, no donor-site morbidity, improved patient safety, and potentially shorter operating times [9]. It can be used with or without bone marrow aspirate and is considered to have excellent moldability [9].

In 2008, Epstein [10] used Vitoss along with laminar autograft and bone marrow aspirate for 60 patients. These patients underwent multilevel laminectomy and noninstrumented posterolateral lumbar fusion (PLF) at one or two levels. The average age of these patients was 70 years. To evaluate the success of the surgeries, the patients were imaged using dynamic X-rays and 2D computed tomography scans at 3–12 months after the operation. Nine (15%) of these patients showed pseudarthrosis [10]. Epstein [11] subsequently compared Vitoss to a nanocrystalline conformation (NanOss) in a study evaluating the use of osteogenic compounds in cervical fusion. Both groups had similar fusion rates; however, one of the shortcomings of Vitoss compared to NanOss was higher pseudoarthrosis rates with deep-wound infections, resulting in graft resorption in the Vitoss group. MacMillan et al. [12] were the first to compare NanOss and Vitoss, demonstrating that NanOss exhibited osteoblastic activity similar to that of normal bone, whereas Vitoss had less osteogenic activity and significantly higher wound infection and pseudoarthrosis rates. NanOss has its own shortcomings as well. NanOss has been contraindicated for use in patients with active metabolic bone diseases, unstable fractures, vascular disease with active wound infections, and patients under immunosuppression or corticosteroid treatments [12].

Research has since focused on enhancing the osteogenic potential of Vitoss by supplementing it with bioactive glass (Vitoss BA), which has been shown to reduce in vivo resorption and enhance the osteogenic differentiation of mesenchymal stem cells [10]. Clinical efficacy has been analyzed for Vitoss as a spinal bone graft extender and in other applications such as the treatment of periodontal, dental, and orthopedic tumor defects [13]. Westhauser et al. studied the utilization of Vitoss and Vitoss BA scaffolds that were preseeded with human mesenchymal stromal cells and subsequently implanted subcutaneously into immunodeficient mice for a duration of 10 weeks [13]. The primary objective of this study included investigating scaffold resorption through the application of microcomputed tomography, assessing osteoid formation and vascularization through histomorphometry and gene expression analysis [14]. The findings revealed that Vitoss BA exhibited a slightly larger propensity for osteoid formation and demonstrated an enhanced capacity for angiogenesis, whereas Vitoss scaffolds exhibited more advanced osteoid maturation. Furthermore, Vitoss implants significantly decreased in volume with a notable increase in the presence of resorbing cells, while Vitoss BA scaffolds exhibited a stable volume. These outcomes underscore the distinctive characteristics and behaviors of Vitoss and Vitoss BA scaffolds when subjected to in vivo conditions [15]. However, future studies are required to unveil the relationship between cells with resorptive activity and bone precursor cells, as the maturation of osteoid tissue increased in the presence of TRAP^+^ cells [15].

### 2.2. Grafton

Grafton (Medtronic, Minneapolis, MN, USA) is a demineralized bone matrix (DBM) product that has both osteoconductive and osteoinductive properties, as demonstrated in an athymic rat assay [16]. It is employed in various orthopedic and reconstructive bone grafting techniques and is available in several forms. The product can be utilized as a bone graft on its own or in conjunction with autologous or other allograft bone in bone grafting techniques. Surfactants, various processing solutions, and antibiotics (gentamicin) in trace levels may be present in this osteobiological material. The allograft tissue undergoes a demineralization process to eliminate factors that could impact the osteoinductivity of the bone matrix [17].

The efficacy of Grafton and similar osteobiological materials is supported by research that shows comparable outcomes to traditional bone grafts, such as iliac crest bone, in applications such as instrumented single-level lumbar fusions [17]. Kang et al. reported on 41 patients, 28 of whom received Grafton DBM treatment while 13 received iliac crest bone graft (ICBG) treatment [17]. After 2 years, the fusion rates were 86% for the Grafton matrix group and 92% for the ICBG group, with no significant statistical difference between groups (p = 1.0). Furthermore, at the 2-year follow-up, the Grafton group demonstrated a slightly better improvement in Oswestry Disability Index (ODI) scores (16.2) compared to the ICBG group (22.7), but that difference did not reach statistical significance (p = 0.2346). At the 24-month mark, the Grafton group continuously demonstrated higher physical function scores; however, these changes did not achieve statistical significance (p = 0.0823) [17].

The study by Kang et al. also assessed intraoperative blood loss. In the case of patients undergoing harvest of the iliac crest, there was significantly greater intraoperative blood loss, being 371 mL greater than that of the individuals in the Grafton group (Grafton: 512 mL; ICBG: 883 mL; p = 0.0031). Apart from the previously mentioned adverse clinical effects associated with harvesting iliac crest bone, it is evident that the blood loss linked to this additional procedure during lumbar fusion surgery should not be underestimated. Cammisa et al. also used Grafton in a randomized clinical trial (RCT) for posterolateral lumbar fusion in 120 patients [18]. Following pedicle screw fixation, they grafted one side with an autograft while the contralateral side was grafted with an autograft and Grafton mixture. Again, the fusion levels were similar between the two sides at the second postoperative year. Vaccaro et al. [19] used Grafton in their prospective study comparing the efficacy and safety of Grafton mixed with aspirated bone marrow or autologous bone in lumbar spinal fusion patients and further demonstrated that the efficacy and safety of Grafton were similar to those of other mixture types.

Conversely, studies investigating the efficacy and safety of Grafton mixed with autologous bone or allografts yielded contradicting results for anterior cervical spinal fusion procedures. An et al. [20] in their RCT reported higher nonunion rates in patients with a mixture of Grafton and allograft compared to allograft alone. Elsawaf et al. [21] and Park et al. [22] used polyether ether ketone (PEEK) cages filled with Grafton and allografts, and autologous bone chips and Grafton, respectively. Both studies showed favorable fusion rates and patient-reported outcomes. The use of Grafton in cervical spinal fusion with allografts or autologous bones warrants further investigation.

Although Grafton has demonstrated efficacy and safety on multiple occasions, the presence of glycerol in the compound has raised suspicions regarding its toxicity [23]. No study has reported glycerol toxicity associated with Grafton use, but authors still suggest cautious use of this osteogenic material.

### 2.3. Mastergraft

The Mastergraft product line (Boat Company, Vonore, TN, USA) consists of resorbable, osteoconductive scaffolds designed to extend autografts or replace bone grafts. These products help keep the patient’s cells viable while facilitating their delivery. Mastergraft technology is offered in various formulations, including freestanding granules, a moldable/packable putty, and a flexible compression-resistant strip [24]. They contain porous granules that can be used alone or in conjunction with bovine type I collagen, creating a biphasic calcium phosphate consisting of 85% B-TCP and 15% HA. The macro- and microporous structure is designed to balance the production of new bone while the material remodels and integrates into the patient’s bone.

In a rabbit posterolateral fusion model, the application of Mastergraft yielded results akin to autograft fusion when assessed radiographically and by manual palpation [24,25]. The group receiving the autograft exhibited a 60% fusion rate in radiographic assessments (6 out of 10 subjects) and a 50% fusion rate in manual palpation assessments (5 out of 10 subjects). In contrast, the group treated with a 50% concentration of Mastergraft Strip showed higher fusion rates of 75% (9 out of 12 subjects) as determined by both radiographic and manual palpation methods. The group treated with a 75% concentration of Mastergraft Strip had slightly lower fusion rates, with 58% (7 out of 12 subjects) showing successful fusion in both radiographic and manual palpation evaluations [24]. Histological analysis revealed an absence of significant adverse inflammatory reactions and there was a notable trend in both Mastergraft Strip groups towards enhanced bone formation across the area of spinal fusion [24]. Although these two studies reported promising results, it is very challenging to translate those results into real-world clinical outcomes and there is no clinical study using Mastergraft as a substitute in the current literature.

### 2.4. SIGNAFUSE

SIGNAFUSE (Bioventus, Durham, NC, USA), available as a putty and in strip format, is a synthetic bone graft composed of bioglass and a biphasic mineral (40% B-TCP and 60% HA). In a recent study, the SIGNAFUSE Strip generated higher levels of osteoblast growth than an earlier synthetic bone graft strip, specifically Actifuse (Baxter, Deerfield, IL, USA) [26]. Actifuse ABX is composed of porous silicate-substituted calcium phosphate, similar to the composition of human bone, and is 100% synthetic, without any biological materials or natural proteins. Actifuse ABX is osteoconductive and osteoinductive [27].

In an established PLF rabbit model, SIGNAFUSE demonstrated better rates of spine fusion and bone remodeling compared to Actifuse ABX when used as a stand-alone synthetic bone graft. At the 6-week mark, the fusion rate achieved through manual palpation was notably higher for SIGNAFUSE (33%) in comparison to Actifuse ABX (0%). However, by 12 weeks, both groups demonstrated an equivalent fusion rate of 50%. When assessing the biomechanical fusion rate using flexion-extension data, it was observed that the SIGNAFUSE group achieved union in 80% of cases, while the Actifuse ABX group had a fusion rate of 44%. Histological analysis of both groups revealed a typical healing response. In all fusion sections under examination, there was a notable presence of moderate neovascularization, fibroconnective tissue, and the development of new bone. It is noteworthy that the degree of new bone formation appeared to be more advanced in the specimens from the 12-week timepoint compared to those from the 6-week timepoint. This suggests a maturation process in bone formation that becomes increasingly evident as the healing period extends [26]. Furthermore, the microcomputed tomography and histomorphometrical data at the 6-week mark showed that the SIGNAFUSE group exhibited a significantly greater degree of new bone formation compared to the Actifuse ABX group. This was demonstrated by a greater degree of structural remodeling and a propensity towards total fusion bed bridging [27].

The strip format of SIGNAFUSE, approved by the FDA for use in 2020, is particularly notable for its rapid hydration and intraoperative handling flexibility. These strips come in various sizes, ranging from 25 to 200 mm, making them suitable for large multilevel procedures. Including bioactive glass in the material has been shown to induce osteoblast differentiation, aiding in bone healing and regeneration [28].

### 2.5. i-FACTOR

A unique anorganic bone matrix (ABM), the i-FACTOR peptide-enhanced bone graft (Cerapedics, Westminster, CO, USA) contains a synthetic 15-amino acid cell-binding peptide (P-15 peptide). Because of its ability to increase cell proliferation, differentiation, and osteogenesis, the FDA first approved this technology in 2015 to aid in the creation of bone in periodontal abnormalities. Interestingly, i-FACTOR does not cause ossification in soft tissues, in contrast to BMP [29]. In a study by Arnold et al. [30], i-FACTOR had statistically better overall clinical success and fusion rates than local autologous bone in patients undergoing anterior cervical discectomy and fusion surgery. It reduced direct and indirect expenditures by achieving fusion rates that were comparable to those of autografts, with a significant increase in overall clinical success [31]. After 1 year, the study successfully met its endpoint, demonstrating that i-FACTOR treatment was not inferior to autograft treatment based on predetermined statistical measures. Successful fusion was achieved for 88.97% of the participants receiving i-FACTOR treatment compared to 85.82% in the autograft group (p = 0.0004). Additionally, neurological success, defined by the authors as maintenance or improvement in neurological function, was comparably high in both groups, with rates of 93.71% in the i-FACTOR group and 93.01% in the autograft group (p < 0.0001) [30].

Arnold et al. [32] subsequently reported the 2-year outcomes of their prospective study, still demonstrating the safety and efficacy of i-FACTOR for single-level degenerative disc disease compared to local autologous bone, although some patients were lost to follow-up.

### 2.6. InQu

Applied as an autograft extender, InQu (Isto Biologics, Hopkinton, MA, USA) is a resorbable osteoconductive poly(lactic-co-glycolic) acid (PLGA) with entangled hyaluronic acid (HyA) intended for use in spine fusion. This extender is designed to minimize the volume of autograft used while maintaining the osteoinductive properties of the biological material. It has been suggested that HyA, a widely dispersed polysaccharide found in bone marrow and the extracellular matrix of connective tissues, is crucial for tissue regeneration and repair [33].

InQu is a synthetic osteobiological product that has been evaluated for its effectiveness and safety in lumbar interbody fusion. Chedid et al. analyzed various outcomes when InQu was used over a 12-month follow-up period, including adverse events, time to fusion, and patient-reported results. They demonstrated that InQu performed better than traditional bone grafts in terms of fusion duration and overall fusion rates over 12 months [33]. The fusion rate of InQu was reported to be quite high; according to a retrospective analysis of 149 spinal fusion procedures, InQu in combination with an autograft demonstrated an overall fusion rate of 93.6% at 12 months [33].

## 3. Discussion

The primary apprehension surrounding synthetic biologics revolves around their safety profile. Concerns stem from several factors that cloud the understanding of their risk-to-benefit ratio. The available data on synthetic osteobiologics is marred by limitations, mainly due to reliance on nonhuman models and small sample sizes, making it difficult to draw definitive conclusions regarding the use of these products in humans. Additionally, a lack of comparative studies further obscures the assessment of their safety, as there is no clearly defined benchmark for comparison [34].

Specific complications associated with synthetic osteobiologics have been documented. Among these, wound complications and heterotopic ossification are noteworthy [35]. Furthermore, the overall level of clinical evidence supporting the safe and effective use of synthetic osteobiologics remains modest.

The decision to utilize specific osteobiological products in spine surgery should be informed by clinical guidelines, patient-specific factors, and the surgical context. Expert opinions and consensus statements from professional societies highlight the importance of individualized treatment planning and suggest that a combination of osteobiological products might offer synergistic benefits in complex cases. Integrating these considerations into clinical practice can enhance decision-making and optimize patient outcomes in spine surgery. Current best practices emphasize the importance of evaluating the type of spinal fusion procedure, patient comorbidities, and previous surgical history when selecting an osteobiological product. For instance, Vitoss is recommended for its osteoconductive properties in treating osseous deformities but requires caution in patients with active metabolic bone diseases or those undergoing immunosuppressive treatments, as seen with NanOss. Additionally, factors such as patient age, bone quality, and comorbid conditions should be considered, with products like i-FACTOR being beneficial for elderly patients with compromised bone healing capacity. Consequently, the potential complications linked to synthetic osteobiologics may exhibit significant variability depending on these contextual factors [36]. Complications including inflammatory response and inadequate bone formation may occur, especially with ceramic-containing synthetic osteobiological materials. In addition, synthetic osteobiological materials containing bioactive glass cause more foreign body reactions and, in some cases, slower absorption rates, which lead to delayed bone formation. Infection risk due to surface properties conducive to bacterial growth and potential toxicity from degradation products can be seen with synthetic osteobiological substances containing polymers. Comparative studies by An et al. [20] and Elsawaf et al. [21] provided valuable insights into the performance of different osteobiologics, suggesting that while Grafton demonstrates efficacy in certain scenarios, its use in anterior cervical spinal fusion warrants further investigation. Case studies illustrating the practical application of these products, such as the favorable outcomes seen with Vitoss in multilevel lumbar fusion, further reveal their potential benefits and limitations. The lack of clinical trials and the challenges of translating in vitro studies to robust clinical outcomes restrict decision-making [35].

The current literature offers further insights into the use of these materials. A recent study from the AO spine group offered a guideline for the use of these materials in anterior cervical discectomy and fusion for spinal degenerative disease [37]. The authors remarked that single use of autografts versus cages with osteobiological materials offered the same level of benefit in terms of patient outcomes. Although autograft use may be associated with donor site morbidity, there is a low certainty level of evidence. The use of allografts versus cages with osteobiologics also offers similar levels of benefit to patients in terms of outcomes. However, the costs for cell-based allografts were relatively higher and that may be an issue for patients with economic disparities. Lambrechts et al., in their recent systematic review assessing the adjunctive role of cellular bone matrices in the promotion of spinal fusion, concluded that routine clinical use is not supported by the current literature due to limited safety [38]. Furthermore, they remarked that the industry’s influence on these products may introduce further biases. Chung et al. compared the efficiency of different osteobiological materials in augmenting anterior cervical fusion [39]. They underscored the disparity in the literature in terms of heterogeneity in study designs and surgical techniques, and no material was able to show superiority compared to others. Hoelen et al. further looked at the use of osteobiologics in patients with multilevel cervical degenerative spine disease undergoing anterior cervical discectomy and fusion and total disc replacement [40]. They underscored the lack of uniform data reporting, insufficient data, and heterogeneity among studies, making it impossible to determine a solid result. This remains a significant limitation in the literature and further long-term comparative studies are needed to show the superiority or benefit of one compound with a high clinical level of evidence.

## 4. Conclusion

The field of bone grafts has experienced remarkable growth, propelled by advancements in materials science and biomedical engineering. This growth is largely due to the enhanced biological attributes of various synthetic products. Materials such as ceramics, glass ceramics, and polymers have proven to be effective as bone graft extenders in spinal fusion procedures, particularly in animal models and some in human studies. They can potentially reduce the use of autografts. A pivotal development in this area has been the advent of 3D printing technology, which has revolutionized the way the microarchitecture of materials can be manipulated. This technological leap has substantially improved both the mechanical strength and the bone healing capabilities of interbody cages and bone graft materials, also allowing for the creation of highly customized and complex scaffold structures for bone regeneration. Bioactive ceramics, such as HA and tricalcium phosphate (TCP), are used in 3D printing to create scaffolds that support bone regeneration. Given the known challenges associated with autograft harvesting or the use of high doses of growth factors, these synthetic products hold great promise in enhancing patient care and surgical outcomes. They offer a potential shift in the approach to bone repair, promising more efficient and less invasive treatments. While acknowledging this promise of synthetic osteobiologics, they do not serve as a substitute for precise surgical techniques. Instead, they should be regarded as valuable adjuncts in bone repair and regeneration.

## Figures and Tables

**Table:** Summary of synthetic biologics used in spine surgery.

Product	Composition/content	Fusion rates (%)	Study context
Vitoss	Beta-tricalcium phosphate (B-TCP)	98.6% at 5.3 months in posterior-lateral noninstrumented lumbar fusion	Human
Grafton	Demineralized bone matrix (DBM)	At 2-year follow-up, Grafton matrix achieved 86% overall fusion while ICGB had 92% fusion	Human
Mastergraft	Biphasic calcium phosphate (85% B-TCP, 15% HA)	75% fusion rate by manual palpation and radiographic scoring (8 weeks)	Animal (rabbit model)
SIGNAFUSE	Bioglass, biphasic mineral (40% B-TCP, 60% HA)	Posterolateral fusion model and fusion rate of 33% at 6 weeks	Animal (rabbit model)
i-FACTOR	Anorganic bone matrix (ABM) with P-15 peptide	Spine fusion rates of 89.7%, 97.3%, and 98.6% at 1, 2, and 6 years	Human
InQu	Poly(lactic-co-glycolic) acid (PLGA) with hyaluronic acid	92.9% at 12 months	Human
